# Combined endoscopic mucosal resection and endoscopic submucosal dissection with an adaptive traction device for a duodenal duplication cyst

**DOI:** 10.1055/a-2522-0276

**Published:** 2025-02-11

**Authors:** Aïmène Khiari, Pierre Lafeuille, Marianne Fricaudet, Florian Rostain, Alexandru Lupu, Jérôme Rivory, Mathieu Pioche

**Affiliations:** 1 Gastroenterology and Endoscopy Unit, Edouard Herriot Hospital, Hospices Civils de Lyon, Lyon, France; 2 Pediatric Hepato Gastroenterology Unit, Femme-Mère-Enfant Hospital, Hospices Civils de Lyon, Lyon, France

Duodenal duplication cysts are rare, accounting for only 2% to 12% of digestive tract duplications 1 . Symptoms typically include abdominal pain, nausea, gastrointestinal bleeding, or pancreatitis 1 . Though generally benign, there is a potential for malignant transformation, making early treatment essential 2 . Surgical management is the standard treatment, although endoscopic marsupialization has been used. A recent study found that only 3 out of 28 patients (10.7%) with intraduodenal cysts without pancreatic or bile duct invasion were treated endoscopically 3 .

We present the case of an 18-year-old patient with intermittent abdominal pain since the age of 2, without blood test abnormalities. Abdominal ultrasound and computed tomography scan revealed a 43 × 27 × 27 mm cyst in the third portion of the duodenum ( Fig. 1 ). Magnetic resonance imaging showed no stones or communication with the bile duct, pancreas, or main pancreatic duct.

**Fig. 1:**
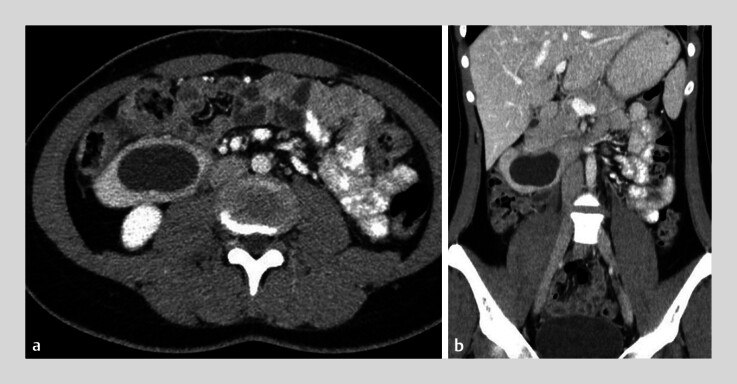
Computed tomography scan of the duodenal duplication cyst. **a** Axial view. **b** Coronal view.

Given these findings, we opted for an endoscopic approach as the first-line treatment. Preoperative endoscopic ultrasound suggested that the duodenal duplication might be confined to the duodenal submucosa. During the procedure, we identified a prolapsing mass within the duodenal lumen, covered with normal mucosa. Using an Olympus PCF 190 TI colonoscope (Olympus, Tokyo, Japan), we performed endoscopic mucosal resection (EMR) to debulk the mass, revealing a pseudo-depressed central area covered with duodenal mucosa. Submucosa was observed on the peripheral sections, prompting us to inject it and proceed with endoscopic submucosal dissection (ESD) using the adaptative traction device A-TRACT (ATRACT Device and Co., Lyon, France) ( Video 1 ) 4 . This technique allowed for precise and controlled removal of the duplicated mucosa, with no residual tissue considering the potential risk of degeneration.

Combined endoscopic mucosal resection and endoscopic submucosal dissection of a duodenal duplication cyst with adaptative traction device.Video 1

The clinical outcome was favorable, with complete resolution of abdominal pain. No immediate or 3-month complications such as bleeding, perforation, or stricture were observed.

Endoscopic management with complete resection using EMR and ESD was effective and safe. It could be a viable option in selected cases.

Endoscopy_UCTN_Code_TTT_1AO_2AG_3AD

## Notes

Endoscopy E-Videos https://eref.thieme.de/e-videos
*E-Videos* is an open access online section of the journal *Endoscopy* , reporting on interesting cases and new techniques in gastroenterological endoscopy. All papers include a high-quality video and are published with a Creative Commons CC-BY license. Endoscopy E-Videos qualify for HINARI discounts and waivers and eligibility is automatically checked during the submission process. We grant 100% waivers to articles whose corresponding authors are based in Group A countries and 50% waivers to those who are based in Group B countries as classified by Research4Life (see: https://www.research4life.org/access/eligibility/ ). This section has its own submission website at https://mc.manuscriptcentral.com/e-videos .
